# Serum S100-Beta as a Biomarker for Neurological Recovery in Acute Spinal Cord Injury (ASCI): A Prospective Case-Control Study

**DOI:** 10.7759/cureus.79202

**Published:** 2025-02-18

**Authors:** Shah Waliullah, Nagendra Kumar, Binod Kumar, Devarshi Rastogi, Zeenat Ara, Uday Raj, Bhavesh Kumar

**Affiliations:** 1 Department of Orthopedic Surgery, King George's Medical University, Lucknow, IND

**Keywords:** acute spinal cord injury (asci), asia scale, biomarker, neurological recovery, s100-beta

## Abstract

Background: Acute spinal cord injury (ASCI) leads to severe neurological deficits with limited prognostic biomarkers. However, S100-beta (S100B), a calcium-binding protein, emerges as a beacon of hope, showing potential as a serological marker of ASCI severity and recovery, inspiring further research and exploration in this field.

Methods: This prospective case-control study included 26 patients with ASCI and 26 age- and sex-matched healthy controls. Serum S100B levels were measured using enzyme-linked immunosorbent assay (ELISA) at baseline, two, and six weeks. Neurological recovery was evaluated using the American Spinal Injury Association (ASIA) Impairment Scale.

Results: Serum S100B levels in ASCI patients were significantly higher than controls at baseline (0.95 ± 0.16 µg/L vs. 0.028 ± 0.02 µg/L; p < 0.05) and at two weeks (p < 0.05). Levels normalized after six weeks (p > 0.05). Also, when comparing serum S100B levels among cases, it was higher in paraplegia than in the paraparesis group. Elevated serum S100B levels correlated with greater injury severity and poorer neurological outcomes.

Conclusion: S100B is a promising biomarker for early ASCI severity and recovery. However, further large-scale studies are required to establish its clinical utility.

## Introduction

Acute spinal cord injury (ASCI) is a debilitating condition with significant morbidity and mortality, affecting neurological function and quality of life. According to WHO, the global incidence of ASCI ranges from 40 to 80 cases per million population annually, whereas in developed nations, 11.5-53.4 per million annual cases of spinal cord injury (SCI) are reported [1-3]. Early diagnosis and timely intervention are crucial in improving outcomes for ASCI patients. Diagnostic tools like clinical examinations and imaging, particularly magnetic resonance imaging (MRI), are indispensable but have limitations in predicting long-term prognosis [4-5].

Biomarkers have gained attention as potential tools to prognosticate and bridge these gaps. S100-beta (S100B) has shown promise as a serologic indicator of central nervous system (CNS) damage and SCI [6]. S100B is a calcium-binding protein predominantly expressed in astrocytes of the CNS [7]. Initially identified as specific to neurons, subsequent studies established its localization in glial cells, regulating intracellular and extracellular calcium metabolism [8].

S100B is released into the bloodstream and cerebrospinal fluid (CSF) following CNS injuries such as traumatic brain injuries, cerebral hemorrhage, and ASCI [9]. Elevated S100B levels are associated with neuronal damage and inflammation, making it a candidate biomarker for assessing injury severity and guiding therapeutic strategies. Importantly, depending on its concentration, it has a dual role in neuroprotection and neurotoxicity. S100B supports neuronal survival and plasticity at low levels but exacerbates inflammation and tissue damage at high concentrations [9].

In ASCI, S100B levels typically rise within hours of the injury and may remain elevated, reflecting the extent of neuronal and glial cell damage [9]. Thelin et al. [10] have explored its diagnostic and prognostic potential in human subjects with traumatic brain injury, with promising findings. Haddadi et al. [11] reported a significant correlation between S100B levels in CSF and the severity of SCI, underscoring its utility in distinguishing complete from incomplete injuries.

Despite its potential, the clinical application of S100B in ASCI remains underexplored, especially in its correlation with prognostication and long-term neurological recovery in human subjects. This study aims to evaluate the prognostic value of serum S100B in ASCI in human subjects, correlating its levels with injury severity and neurological recovery.

## Materials and methods

This prospective case-control study was conducted at King Georges Medical University, Lucknow, India, over one year after obtaining institutional ethical clearance (XVI-PGTSC-IIA/P75).

The study sample consisted of 26 cases of traumatic acute spinal cord injury (TASCI). The sample size is determined using the following formula [12]: $N = \frac{(Z_{a/2})^2 P (1 - P)}{d^2}$ where N represents sample size, P represents prevalence, a represents the error, d represents the degree of freedom, and Z_a/2_ represents the differentiation coefficient (1.96 or 2).

Based on Srivastava et al. (2015) [13], the prevalence of ASCI was reported as 6% (p = 0.06). Substituting these values, the calculated sample size was 23. To account for potential follow-up losses, three additional cases were included, bringing the final sample size to 26 cases. Thus, the study included 26 TASCI subjects and 26 healthy controls, making a total of 52 participants.

Participants were divided into two groups of 26 cases (aged 18-60 years, American Spinal Injury Association (ASIA) A-D) and 26 controls (healthy, age- and sex-matched individuals). Cases with spinal fractures admitted within 24 hours of trauma with informed consent were included in the study. Cases with associated head injury, abdominal injury, non-traumatic SCI, pathological spinal fracture, and polytrauma were excluded from the study. Patients were not given any steroids after admission.

Serum samples were collected at three time points: baseline (within 24 hours of injury), two weeks, and six weeks post-injury. Blood samples were drawn via venipuncture. All samples were processed immediately, centrifuged, and stored at -80°C for analysis.

The concentrations of S100B in serum were quantified in micrograms per liter (µg/L). Neurological function was evaluated using the ASIA Impairment Scale. Based on sensory and motor function recovery, patients were categorized into complete injury (ASIA grade A) and incomplete injuries (grades B, C, and D).

Statistical analysis

Data were analyzed through IBM SPSS Statistics for Windows, Version 25 (Released 2017; IBM Corp., Armonk, New York, United States), and comparisons between the ASCI (paraplegia and paraparesis) group and healthy controls were performed using various biostatistics tools. A p-value less than 0.05 was considered statistically significant.

For comparing serum S100B levels between two groups (e.g., ASIA A vs. ASIA BCD), a t-test was used for normal distribution. For comparing serum S100B levels across multiple groups (e.g., ASIA A, B, C, and D), ANOVA was used for normal distribution. For comparing categorical variables (e.g., gender, injury type), the chi-square test was used. A comparison of serum S100 levels in cases and controls was performed using a t-test for both groups.

## Results

A total of 26 patients with a mean age of 30.4 ± 10.3 years, with 84.6% males, were enrolled. Most injuries resulted from falls (50%) and road traffic accidents (42.3%). Out of 26 patients, 15 had complete paraplegia (ASIA A), while 11 had incomplete injuries and paraparesis (ASIA BCD). S100B protein levels in serum were measured using enzyme-linked immunosorbent assay (ELISA) kits (E-EL-H1297).

Comparison of serum S100 levels in cases and controls

**Figure 1:**
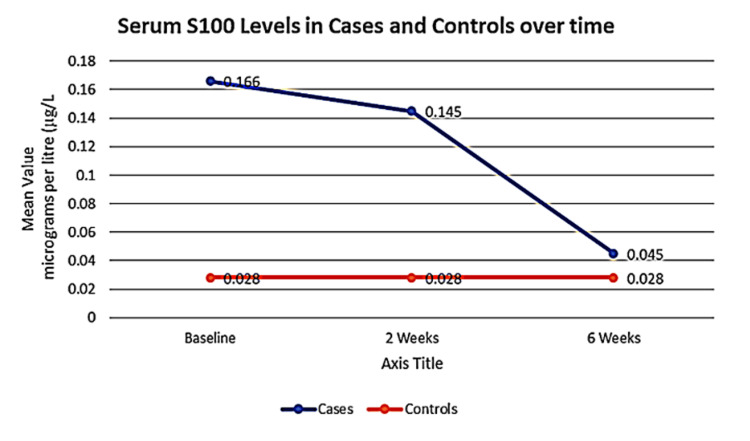
Serum S100 Levels in cases and controls over time

Cases had significantly higher levels (0.95 ± 0.16 µg/L) than controls (0.028 ± 0.02 µg/L; p < 0.05) at baseline, and levels remained elevated in cases (p < 0.05) at two weeks. At six weeks, levels declined and showed no significant difference between groups (p > 0.05) (Table 1, Figure 1).

**Table 1:** Serum S100 levels in cases and controls over time

Serum S100	Cases (n = 26)	Controls (n = 26)	p-value
Baseline	0.166 ± 0.016	0.028 ± 0.02	0.025
Two weeks	0.145 ± 0.017	0.028 ± 0.02	0.028
Six weeks	0.045 ± 0.030	0.028 ± 0.02	0.637

Comparison of serum S100 levels in cases and their comparison between paraplegia and paraparesis

**Figure 2:**
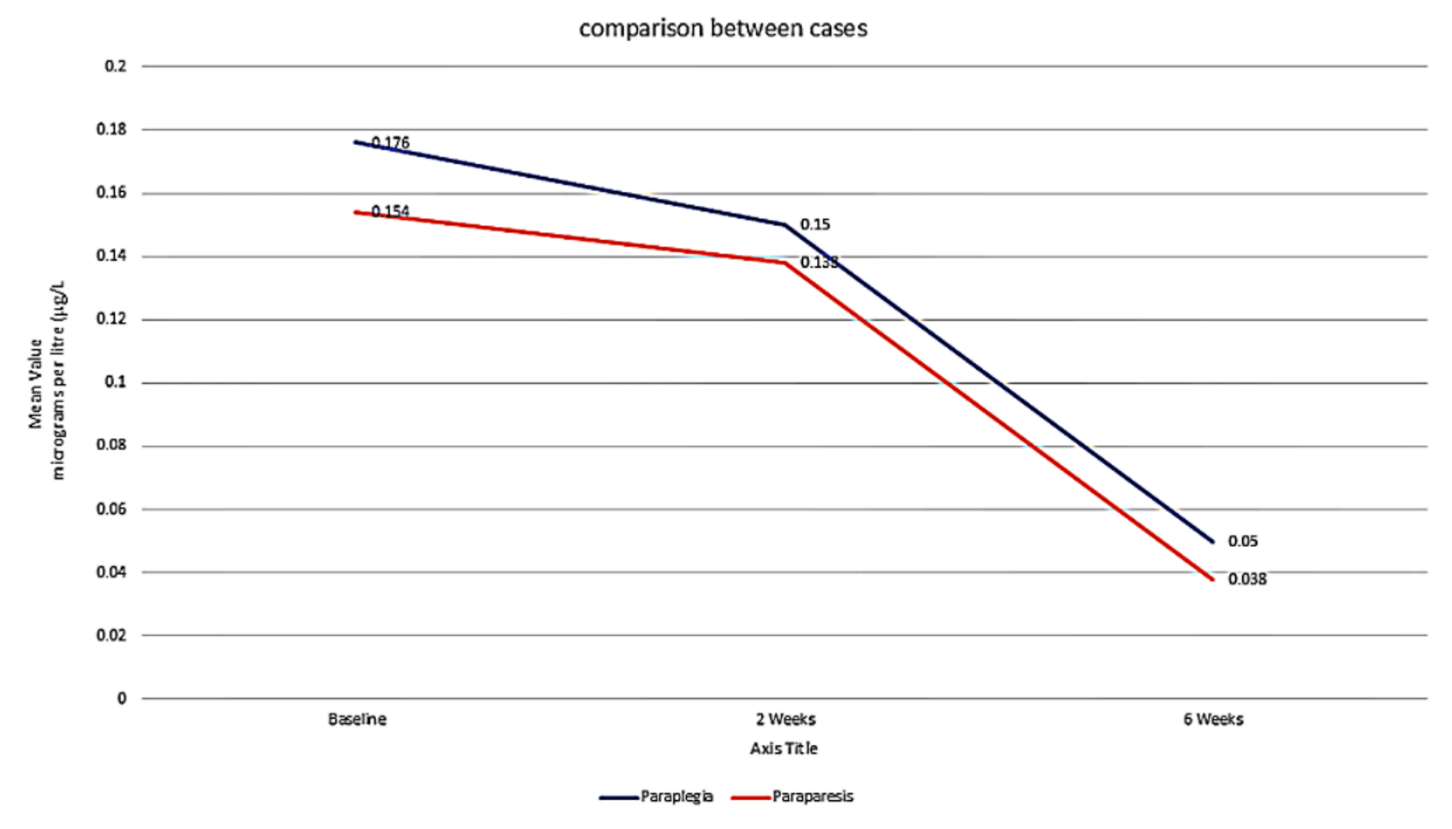
Comparative serum S100 levels in cases with paraplegia and paraparesis

On further analyzing the variation of serum S100 levels in cases, the serum S100B level in the case of paraplegia was higher than in the case of paraparesis across baseline, two weeks, and six weeks. The association of serum S100 values in cases and controls was found to be statistically significant at baseline and two weeks (p < 0.05), but it was non-significant at six weeks (p > 0.05) (Table 2, Figure 2). The rate of decline of serum S100 level was higher in paraparesis than in paraplegia. Elevated S100B levels correlated with greater injury severity and poorer neurological outcomes.

**Table 2:** Serum S100 levels in cases and their comparison between paraplegia and paraparesis

Serum S100	Paraplegia (15)	Paraparesis (11)		Total cases (26)	p-value
Baseline	0.176	0.154	0.166		<0.05
Two weeks	0.150	0.138	0.145		<0.05
Six weeks	0.050	0.038	0.045		>0.05

## Discussion

The pathophysiology of ASCI involves primary mechanical insult to the spinal cord and a secondary cascade of cellular events involving inflammation, oxidative stress, and cellular apoptosis, which exacerbates the initial injury. Inflammation is particularly significant, contributing to the progressive damage observed in ASCI [11]. Although traditional diagnostic tools, including neurological examination, imaging, and electrophysiological studies, are indispensable, they do not quantify the extent of tissue damage or provide insights into prognosis. Biomarkers are biomolecules released in blood and CSF after a breach of the blood-spinal cord barrier (BSCB) after spinal cord trauma [14].

S100B has emerged as a promising biomarker for assessing traumatic brain and ASCI severity [14-15]. Its utility in brain injuries and stroke has been well established, but its role in ASCI remains underexplored [16]. Previous studies support the role of S100B as a marker of spinal cord damage. Lee et al. [16] and Undén et al. [17] reported elevated S100B levels in patients with vertebral fractures, even without neurological involvement, suggesting extracerebral sources such as damaged vertebrae. Marquardt et al. [18] further postulated that spinal fractures release S100B into the bloodstream, with levels increasing significantly during spinal cord involvement. These findings suggest that S100B serves as a biomarker not only for neuronal damage but also for overall spinal trauma.

Serum S100B levels in our study were significantly elevated in ASCI patients compared to controls at baseline and remained elevated at two weeks. By six weeks, levels had normalized, with no statistically significant difference between cases and controls. This trend mirrors findings by Wolf et al. [19], which demonstrated that elevated S100B levels correlate with ASCI severity, particularly in patients with neurological deficits.

Serum S100B levels were significantly higher in patients with paraplegia compared to those with paraparesis at initially measured intervals of up to two weeks, reflecting the greater severity of neurological damage in complete injuries. The rate of decline in S100B levels was steeper in paraparesis patients than in paraplegia cases. This aligns with findings by Wolf et al. [19] and Munce et al. [20], who observed that more severe injuries exhibited prolonged elevations in S100B due to sustained inflammatory and cellular damage.

Statistical analysis revealed a significant difference in S100B levels between cases and controls at two weeks, consistent with the ongoing inflammatory response. By six weeks, S100B levels in ASCI cases had normalized, and the difference compared to controls was no longer statistically significant, indicating subsidence of the inflammatory state.

Haddadi et al. [11] highlighted that serum measurements may have reduced sensitivity compared to CSF assessments due to dilution effects in peripheral circulation. In our study, we measured only serum levels of S100B and observed a significant correlation with serum levels. So, serum evaluation can be a practical, non-invasive alternative for CSF evaluation in monitoring ASCI severity with S100B. Serum evaluation is less time-consuming, inexpensive, and helpful in resource-limited settings.

This study provides strong evidence to support the fact that, in patients with spinal cord injuries and suspected neurological deficits, S100B can serve as a potential biomarker for the estimation of injury severity. However, our results cannot be generalized for all patients with spinal fractures. This study includes patients with thoracolumbar fractures; further inclusion of cervical patients may provide more insight into variation in S100B levels. The results cannot be applied directly in a clinical setting because our study population was small, and the duration of follow-up was small. Future studies with larger cohorts and multicenter designs with more extensive follow-up are needed to validate these findings.

## Conclusions

This study highlights the promising role of serum S100B as a crucial biomarker for assessing both the severity of ASCI and the subsequent recovery process. The fluctuations in serum S100B levels observed over time highlight its dynamic characteristics, positioning it as an invaluable tool for prognosis and ongoing monitoring of patient recovery. By integrating S100B into clinical protocols, healthcare providers could significantly enhance early decision-making processes and enable more tailored treatment strategies for individuals grappling with the challenges of ASCI.
